# Complete genome of five *Acinetobacter baumannii* phages

**DOI:** 10.1128/mra.00463-25

**Published:** 2025-07-11

**Authors:** Martina Scarrone, Dann Turner, Johannes Wittmann, Christine Rohde, Simon J. Labrie, Denise M. Tremblay, Sylvain Moineau

**Affiliations:** 1 Département de biochimie, de microbiologie et de bio-informatique, Faculté des sciences et de génie, Université Laval 98637 , Quebec City, Quebec, Canada; 2 School of Applied Sciences, College of Health, Science and Society, University of the West of England 152370, Bristol, United Kingdom; 3 Leibniz Institute DSMZ-German Collection of Microorganisms and Cell Cultures GmbH 28351, Braunschweig, Germany; 4 SyntBioLab Inc., Lévis, Quebec, Canada; 5 Félix d’Hérelle Reference Center for Bacterial Viruses, Faculté de Médecine Dentaire, Université Laval 70345 , Quebec City, Quebec, Canada; Queens College Department of Biology, Queens, New York, USA

**Keywords:** bacteriophage, Acinetobacter

## Abstract

*Acinetobacter baumannii* tailed phages Bhz9, Bhz15, Bhz16, Bhz20, and Bhz21 were isolated from wastewater samples in Hamburg-Eppendorf (Germany). Their double-stranded DNA genomes range from 41,054 to 167,277 bp, encoding between 54 and 267 predicted genes, with a G + C content ranging from 36.4% to 48.9%. Only phage Bhz15 encodes tRNAs.

## ANNOUNCEMENT

Phages were isolated from 1 L of wastewater from Hamburg-Eppendorf in Germany (coordinates: 53.59001723110464 and 9.978468493378452). Filtered samples (0.45 µm; Sartorius, Germany) were added to *Acinetobacter baumannii* clinical strains (Table 1) in Tryptic Soy Agar (TSA) medium and incubated at 37°C for 18 h. Plaques were isolated using the double-agar layer method and amplified with their host in TSA at 37°C for 18 h (1). Transmission electron microscopy observations revealed three morphotypes: siphoviruses (Bhz9 and Bhz20), podoviruses (Bhz16 and Bhz21), and myoviruses (Bhz15) (2).

**TABLE 1:** Genomic description of the five *A. baumannii* phages

Phage	Host strain	Genome length (bp)	GC^*a*^ (%)	Genome coverage (*x*)	No. of reads	No. of *orfs*	GenBank accession no.	SRA accession no.
Bhz9	DSM 106838	43,686	48.2	50.5	1,335,261	66	PV067731	SRR32578190
Bhz15	DSM 25645	167,277	36.4	25.1	789,343	267	PV067732	SRR32578189
Bhz16	DSM 25645	41,732	39.3	170.5	222,545	60	PV067733	SRR32578188
Bhz20	DSM 106838	43,452	48.9	214.0	177,489	68	PV067734	SRR32578187
Bhz21	DSM 25645	41,054	40.3	188.5	220,631	53	PV067735	SRR32578186

^*a*^ GC, guanine and cytosine.

Phage genomic DNA was extracted from high-titer lysates (>10⁹ PFU/mL) following a phenol-chloroform protocol (3).Libraries were prepared with the Illumina DNA Prep kit and Integrated DNA Technologies 10 bp unique dual indexes and sequenced on NextSeq 2000 (2 × 151 bp; SeqCenter, Pittsburgh, USA). Reads were processed with bcl-convert (v.4.1.5), while assemblies were generated using SPAdes (v.3.13.0) (4) and Ray (v.3.0.1-rc) (5), selecting the best coverage results. Quality control and adapter removal were done with Trimmomatic (v.0.39) (6). Genome completeness was assessed with CheckV (v.1.0.3) (7), and genome ends were confirmed by Sanger sequencing. Gene calling was performed using the phold pipeline (8–13) with additional functional inference obtained using InterProScan (14) and HHsuite (15). Related phage genomes were identified using BLASTn limited to *Caudoviricetes* and excluding *Caudoviricetes* sp. Genome maps were produced using clinker (16). Default parameters were used for all software.

**Fig 1:**
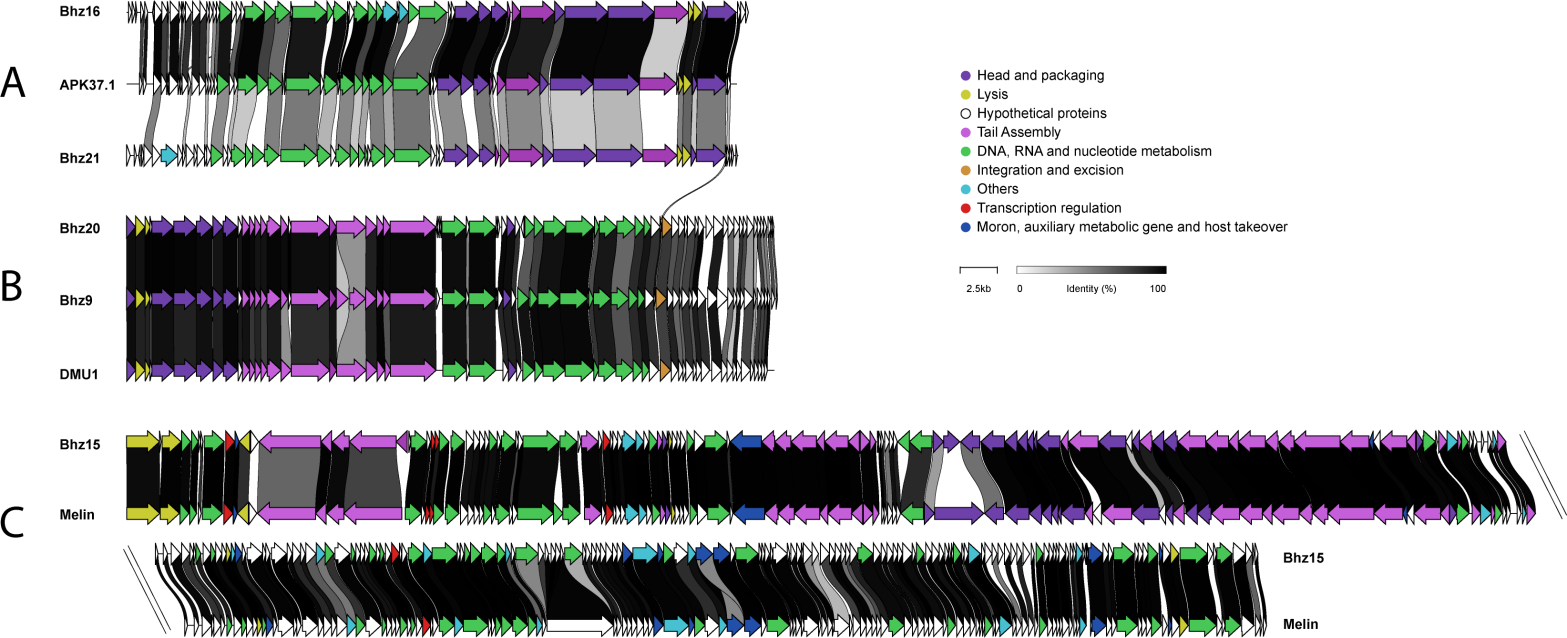
Genome alignment of the five *A. baumannii* phages and their closest relatives. (**A**) Bhz16 and its relative phage, APK37.1 (*Friunavirus* genus, accession number MZ967493.1), with phage Bhz21 as a distant relative; (**B**) Bhz9 and Bhz20 and their relative phage, DMU1 (unclassified *Caudoviricetes*, accession number MT992243.1); and (C) Bhz15 and its relative phage, Melin (*Straboviridae* family, accession number MW388003.1). The identity regions in amino acids are represented in shades of gray. The functional genomic modules are color-coded.

The podoviruses Bhz16 and Bhz21 have double-stranded DNA genomes of 41,732 and 41,054 bp, respectively, with a guanine and cytosine (GC) content ranging from 39% to 40% (Table 1). Both were sequenced at a coverage depth exceeding 170×. These strictly lytic podoviruses encode 60 and 53 predicted open reading frames (*orfs*), respectively. Comparative genome analysis indicated that they belong to the genus *Friunavirus*. The genome of Bhz16 showed 89% nucleotide identity to that of *Acinetobacter* phage APK37.1, while Bhz21 was more distantly related (Fig. 1A). To facilitate comparison with other *Friunavirus* genomes, the genomes were reopened at the terminal repeat.

Siphoviruses, Bhz9 (43,686 bp) and Bhz20 (43,452 bp), encode 66 and 68 *orfs*, respectively, with a GC content of 48% (Table 1). Their sequencing coverage was over 50× and 214×. They share 97% nucleotide identity with *Acinetobacter* phage DMU1 (Fig. 1B) and exhibit features of a temperate lifestyle (integrase). To maintain consistency with their closest relative, their genomes were opened at the small terminase subunit.

Phage Bhz15 has a large genome (167,277 bp, 36.4% GC, 267 *orfs*) obtained from 25× coverage (Table 1). It is a distinct T4-like lytic myovirus related to members of the *Straboviridae* family, such as *Acinetobacter* phage Melin as its closest relative (96% identity). It contains genes involved in DNA replication, host interaction, and structural protein formation. It also encodes six tRNA genes, potentially countering host tRNA depletion or compensating GC content disparities to enhance translation efficiency during infection (17). Its genome was opened at the *rIIA* gene to align with other *Straboviridae* phages (Fig. 1C).

These phages are available through DSMZ (Germany) and the Félix d’Hérelle Reference Center for Bacterial Viruses (Université Laval, Canada).

## Data Availability

Whole genome sequences were submitted to the National Center for Biotechnology Information as BioProject PRJNA1194496. Accession numbers for both GenBank and Sequence Read Archive are listed in Table 1.
